# The Use of an Adjuvant System Improves Innate and Adaptive Immune Response When Associated with a *Leishmania* (*Viannia*) *braziliensis* Antigen in a Vaccine Candidate against *L.* (*Leishmania*) *infantum* Infection

**DOI:** 10.3390/vaccines11020395

**Published:** 2023-02-09

**Authors:** Fernando Augusto Siqueira Mathias, Thais Lopes Valentim Di Paschoale Ostolin, Levi Eduardo Soares Reis, Jamille Mirelle de Oliveira Cardoso, Rory Cristiane Fortes De Brito, Rodrigo Dian de Oliveira Aguiar Soares, Bruno Mendes Roatt, Paula Melo de Abreu Vieira, Alexandre Barbosa Reis

**Affiliations:** 1 Laboratório de Imunopatologia, Núcleo de Pesquisas em Ciências Biológicas/NUPEB, Universidade Federal de Ouro Preto, Ouro Preto 35400-000, Minas Gerais, Brazil; 2 Departamento de Análises Clínicas, Escola de Farmácia, Universidade Federal de Ouro Preto, Ouro Preto 35400-000, Minas Gerais, Brazil; 3 Laboratório Multiusuário de Citometria de Fluxo, Núcleo de Pesquisas em Ciências Biológicas/NUPEB, Universidade Federal de Ouro Preto, Ouro Preto 35400-000, Minas Gerais, Brazil; 4 Instituto Nacional de Ciência e Tecnologia em Doenças Tropicais (INCT-DT), Salvador 41500-290, Bahia, Brazil; 5 Laboratório de Morfopatologia, Núcleo de Pesquisas em Ciências Biológicas/NUPEB, Universidade Federal de Ouro Preto, Ouro Preto 35400-000, Minas Gerais, Brazil; 6 Departamento de Parasitologia, Instituto de Ciências Biológicas, Universidade Federal de Minas Gerais, Belo Horizonte 31270-901, Minas Gerais, Brazil

**Keywords:** adjuvants, adjuvant system, saponin, monophosphoryl lipid A, resiquimod, cell recruitment, memory T cell, parasite load, vaccine

## Abstract

Background: The adjuvants’ optimal dose and the administration route can directly influence the epitope recognition patterns and profiles of innate response. We aimed to establish the effect and the optimal dose of adjuvant systems for proposing a vaccine candidate to be employed with *Leishmania* (*Viannia*) *braziliensis*. Methods: We evaluated the adjuvants saponin (SAP), monophosphoryl lipid A (MPL) and resiquimod (R-848) isolated and combined as adjuvant systems in a lower dose corresponding to 25%, 33%, and 50% of each adjuvant total dose. Male outbred BALB/c mice were divided into 13 groups, SAP, MPL, and R-848 isolated, and the adjuvant systems SAP plus MPL (SM), SAP plus R-848 (SR), and MPL plus R-848 (MR). Results: SM50 increased levels of all chemokines analyzed and TNF production, while it presented an increased inflammatory cell infiltrate in the skin with macrophage recruitment. Thus, we proposed a vaccine candidate employing *L.* (*V.*) *braziliensis* antigen associated with the SM adjuvant system against experimental *L.* (*Leishmania*) *infantum* challenge. We observed a significant increase in the frequency of cells expressing the central and effector memory CD4^+^ T cells phenotype in immunized mice with the LBSM50. In the liver, there was a decreased parasite load when mice received LBSM50. Conclusions: When combined with *L.* (*V.*) *braziliensis* antigen, SM50 increases TNF and IFN-γ, which generates central and effector memory CD4^+^ T cells. Therefore, using an adjuvant system can promote an effective innate immune response with the potential to compose future vaccines.

## 1. Introduction

Parasitic diseases (i.e., leishmaniasis, malaria, schistosomiasis, hookworm, and Chagas disease) as well as other neglected diseases affect a large fraction of worldwide population leading to a significant increase in morbidity and mortality [1]. Vaccination is one of the most efficient, safe, and cost-efficient public health actions for controlling and even eradicating infectious diseases with a medium- and long-term capability for reducing morbidity and mortality [2,3,4]. Despite many vaccines being successfully effective against a broad range of relevant pathogens, an efficient and protective vaccine against these neglected diseases is still required [2]. For both canine and human leishmaniasis, several vaccines were investigated, but with just a few promising results that compromise their prophylactic use [5,6].

Developing of prophylactic vaccines against complex pathogens requires a multifaceted immune response activation for infection prevention, which, until recently, unsuccessfully achieved using the available technologies [2]. An attempt to overcome this challenge relies on recombinant proteins and peptide antigens, but often needs the addition of adjuvants to potentiate the immune response after vaccination [7,8].

Adjuvants are widely used to create more efficient and protective vaccines since their combination with antigens potentiate components of innate immunity, leading to an adaptive immune response that includes humoral and cell-mediated responses [7,9,10]. Nowadays, the adjuvant system (AS) is raised as a combination between immunostimulatory molecules designed to allow vaccines to provide a better and broader protection than classical formulations [11,12,13].

Choosing the immunization route is also a crucial aspect that directly influences the vaccine success. Several studies show that the intradermal route has a good performance and efficiency for stimulating the immune system, as well as other usual routes easily accessed [14,15,16,17].

Given this scenario, we choose to investigate systems composed of double associations between saponin (SAP), monophosphoryl lipid A (MPL), and resiquimod (R-848) adjuvants via intradermal route. These adjuvants were selected based on their action mechanism [18]. Saponins are natural triterpenoid and steroidal glycosides that exhibit many different biological actions, such as stimulating T cells, making their use interesting in subunit vaccines against intracellular pathogens [19,20,21]. The MPL adjuvant is a detoxified derivate from LPS and was evaluated already in a large number of human clinical trials and in several vaccines or phase III trials, such as Fendrix^®^ (Hepatitis B, GlaxoSmithKline UK), Cervarix^®^ (human papillomavirus-16 and -18 (HPV 16/18), GlaxoSmithKline UK), Pollinex^®^ Quattro (Tree-/Grass Pollen, Allergy Therapeutics UK), and RTS,S/AS01 vaccine (Mosquirix™) GlaxoSmithKline UK RTS,S^®^ (malaria) [22,23,24]. It is known that MPL induces an immune response in various immune cell types, i.e., mouse epithelial cells, dendritic cells (mDCs), B and T cells, among others [7,25]. As imiquimod, R-848 adjuvant is part of imidazoquinoline family and a Toll-like receptor (TLR) 7 and 8 agonist [26,27]. In addition to being effective to stimulate adaptive immunity, R-848 was already tested as a treatment for *Leishmania (Leishmania) infantum* in mice with systemic immunomodulation and parasite burden reduction [28].

Therefore, we aimed to investigate whether an adjuvant system using a reduced dose is able to induce, at least, an equal innate immune response in the BALB/c mouse skin when compared to the total dose of each adjuvant alone. Furthermore, we combined the best adjuvant system with the crude antigen of *L*. (*V*.) *braziliensis* and challenged the immunized animals with *L*. (*L*.) *infantum* to evaluate the efficacy of this vaccine candidate in generating memory cells and, hence, reducing parasite load.

## 2. Materials and Methods

### 2.1. Ethics Statement

All procedures were performed according to the Brazilian National Animal Care Ethical Council (CONCEA). This study was approved by the Ethical Committee on the Use of Animals (CEUA) of the Federal University of Ouro Preto (UFOP) (protocol number 2014/11). Male outbred BALB/c mice (6 to 8 weeks old) were maintained in the Animal Science Center facility at UFOP, Minas Gerais, Brazil.

### 2.2. Mice and Skin Sensitization

The animals were divided into 13 groups (*n* = 12/group): (1) control group received sterile saline 0.9% (NaCl); (2) SAP group received a 100 μg of saponin; (3) MPL group received a 50 μg of monophosphoryl lipid A; (4) R-848 group received a 25 μg of resiquimod; (5) SM25 group received a 25 μg of saponin and 12.5 μg of MPL; (6) SM33 group received a 33 μg of saponin and 16.5 μg of MPL; (7) SM50 group received a 50 μg of saponin and 25 μg of MPL; (8) SR25 group received a 25 μg of saponin and 6.25 μg of R-848; (9) SR33 group received a 33 μg of saponin and 8.25μg of R-848; (10) SR50 group received a 50 μg of saponin and 12.5 μg of R-848; (11) MR25 group received a 12.5 μg of MPL and 6.25 μg of R-848; (12) MR33 group received a 16.5 μg of MPL and 8.25 μg of R-848; (13) MR50 group received a 25 μg of MPL and 12.5 μg of R-848. Saponin (Sigma Chemical Co., St. Louis, MO, USA) and resiquimod (Sigma Chemical Co., St. Louis, MO, USA) were prepared in sterile saline at a concentration of 1 mg/mL and 5 mg/mL, respectively, and monophosphoryl lipid A (Avanti Polar Lipids, Inc., Alabaster, AL, USA) was resuspended at a concentration of 2 mg/mL with a solution of *Bovine Serum Albumin* (BSA) according to the manufacturer’s instructions. The adjuvant system groups received a lower dose combination of two adjuvants corresponding to 25%, 33%, and 50% of each adjuvant total dose. The animals received a total volume of 50 μL of each adjuvant isolated or the adjuvant system by an intradermal route at the dorsum [7,29]. After 48 h of sensitization, skin samples from the inoculum site were collected for cell recruitment and inflammatory cells infiltration evaluation, and chemokines (CCL2, CCL3, CCL4, CCL5, and CXCL1) and cytokines (IL-2, TNF, IFN-γ, and IL-10) measurement.

### 2.3. Cell Recruitment Profile and Differential Leukocyte Counts

Skin samples were preserved in methanol/dimethyl sulfoxide (DMSO) (4:1), dehydrated, and embedded in paraffin. Posteriorly, they were cut by microtome into (5 μm) sections, deposited on a slide, and stained with hematoxylin–eosin (HE) for quantitative and differential analysis of the cell recruitment profile. The slide was visualized by the 40× objective and 20 random images (total area covered 1.5 × 106 μm^2^) were digitized using a microcamera Leica DM5000B attached to a microscope and Leica Application Suite program (version 2.4.0 R1 Leica Microsystems-Switzerland-Ltd, Heerbrugg, Switzerland). To quantify inflammatory cell infiltrates in the dermis/epidermis the images were analyzed using the Leica QWin V3 program (Leica Microsystems-Switzerland-Ltd, Heerbrugg, Switzerland) for counting all cell nuclei, excluding hair follicles from the analysis. The results are expressed by the number of cells per 75,256.2 mm^2^. To identify the cell recruitment profile to the inoculum site, we performed a differential leukocyte count (mononuclear or polymorphonuclear) using optical microscopy. The results are expressed by the percentage values of cells infiltrating differential leukocyte count and displayed on pie charts.

### 2.4. Chemokines and Cytokines Measurement

Skin samples received 500 μL of inhibitor protease (Sigma Chemical Co., St. Louis, MO, USA) and were homogenized in a TissueLyser II (Qiagen, Hilden, Germany). Then, the homogenate samples were centrifuged at 10,000× *g* for 10 min at 4 °C and the supernatants were collected. The chemokines CCL2, CCL3, CCL4, CCL5, and CXCL1 were measured using a BD^TM^ CBA Flex Set assays (BD Biosciences, San Jose, CA, USA) according to the manufacturer’s instructions. The cytokines IL-2, TNF, IFN-γ, and IL-10 were measured by BD^TM^ Cytometric Bead Array (CBA) Mouse Th1/Th2/Th17 Cytokine Kit (BD Biosciences, San Jose, CA, USA) according to the method as described previously [7]. The events were acquired on the FACSCalibur cytometer (BD Biosciences, San Jose, CA, USA). The results were obtained using the FCAP Array^TM^ v1.0.2 software (BD Biosciences, San Jose, CA, USA) and expressed by picograms per milliliter (pg/mL) of total protein.

### 2.5. Mice Immunization and Challenge

After assessing the effect of the adjuvant system in the mouse skin and establishing its optimal dose, we proposed a vaccine candidate employing *Leishmania* (*V.*) *braziliensis* antigen associated with the best adjuvant system against experimental challenge in a murine model. Thus, the animals were divided into four groups (*n* = 10/group): (1) SAL received sterile saline 0.9% (NaCl 0.9%, pH 7.2–7.4); (2) LB received 60 μg of *L.* (*V.*) *braziliensis* antigen; (3) SM50 adjuvant group received 50 μg of saponin plus 25 μg of monophosphoryl lipid A; and (4) LBSM50 received 60 μg of *L.* (*V.*) *braziliensis* antigen and 50 μg of saponin plus 25 μg of monophosphoryl lipid A. The mice received three intradermal immunizations in the dorsum, with two-week intervals between doses. After two weeks, the mice were challenged with a 1 × 10^7^ stationary phase *Leishmania* (*L.*) *infantum* promastigotes via tail vein. Twenty-eight days post-challenge, spleen samples were collected for evaluation of T cell proliferation (CD3^+^CD4^+^ and CD3^+^CD8^+^), intracellular cytokine production (TNF, IFN-γ, and IL-2), central and effector memory T cells (T_CM_ and T_EM_), and quantification of parasite load, and liver samples were collected for quantification of parasite load.

### 2.6. Leishmania (V.) braziliensis Antigen

The *L.* (*V.*) *braziliensis* strain (MHOM/BR/75/M2903) was cultured in medium *Novy-MacNeal-Nicolle*/*Liver Infusion Tryptose* (NNN/LIT). The antigen was prepared as described by [30] and stored at −80 °C until use. Briefly, promastigote disruption was performed using ultrasound (Branson Sonifier^®^ Ultrasonic cell disruptor) with five cycles of one min at 40 watts at fifteen seconds intervals.

### 2.7. Parasites and Soluble Leishmania (L.) infantum Antigen

The *L.* (*L.*) *infantum* (MCAN/BR/2008/OP46) promastigotes were cultured at 24 °C in medium NNN/LIT supplemented with fetal bovine serum (FBS) 10%. Stationary growth parasites were resuspended in PBS supplemented with FBS 3% to infect mice and also to prepare the soluble *L.* (*L.*) *infantum* antigen (SLiAg) [31].

### 2.8. Lymphocytes Proliferation Assay and Intracellular Cytokines Staining

The T lymphocyte proliferation was estimated by *Carboxyfluorescein succinimidyl ester* (CFSE) as described previously [32]. Total splenocytes were plated at 5 × 10^5^ cells in 96-well round bottom (Costar) and stimulated culture with SLiAg (50 μg/mL) for 5 days at 37 °C with 5% CO_2_. Cells were stained with Fixable Viability Stain 780, BD Horizon^TM^ (FVS780) at room temperature for 15 min. Cells were washed and treated with PBS and an inert protein (serum albumin 5%). Cells were stained with monoclonal antibodies against CD3 BV650 (clone 145.2C11, BD Horizon^TM^), CD4 BV605 (clone RM4-5, BD Horizon^TM^), and CD8α BV786 (clone 53–6.7, BD Horizon^TM^) at room temperature for 30 min. Cells were fixed with FACS fixing solution (10 g/L paraformaldehyde, 10.2 g/L sodium cacodylate, and 6.63 g/L sodium chloride, pH 7.2) and washed. Cells were permeabilized and stained with antibodies against TNF-α PE-Cy7 (clone LG.3A10, BD Pharmingen^TM^), IFN-γ AF700 (clone XMG1.2, BD Pharmingen^TM^), and IL-2 PE (clone JES6-5H4, BD Pharmingen^TM^) cytokines at room temperature for 30 min. The events (300,000) were acquired on the LSRFortessa^TM^ cytometer (BD Biosciences, San Jose, CA, USA) using FACSDiva software. Data were analyzed using the FlowJo^TM^ software (BD Biosciences, San Jose, CA, USA). The results are expressed by the index obtained by the SLiAg-stimulated culture/unstimulated culture ratio for T lymphocyte proliferation and by the frequency of the SLiAg-stimulated culture for intracellular cytokine production.

### 2.9. Central and Effector Memory T Cell Phenotypes

Central and effector memory T cells were assessed twenty-eight days post-challenge, as previously described [33]. Total splenocytes were plated at 5 × 10^5^ cells in a 96-well round-bottom (Costar) and stimulated with SLA (50 μg/mL) at 37 °C with 5% CO_2_. Past 5 days of antigen-specific stimulation, samples were blocked with anti-mouse CD16/CD32 (BD Pharmingen^TM^) (0.5 μg/well) and stained with markers related to memory T cells phenotype, anti-mouse CD3 FITC (clone 17A2, BD Pharmingen^TM^), anti-mouse CD4 BV605 (clone RM4-5, BD Horizon^TM^), anti-mouse CD8α PerCPCy5.5 (clone 53-6.7, BD Pharmingen^TM^), anti-mouse CD44 APC (clone IM7, BD Pharmingen^TM^), anti-mouse CD45RA BV711 (clone 14.8, BD Pharmingen^TM^), anti-mouse CD62L AF700 (clone MEL-14, BD Pharmingen^TM^), anti-mouse CD127 BV510 (clone SB/199, BD Horizon^TM^), and anti-mouse CD197 BV421 (clone 4B12, BD Horizon^TM^). The results are expressed by the frequency of the SLiAg-stimulated culture.

### 2.10. Parasite Load in Mice Spleen and Liver

To determine the parasite load in the spleen and liver of mice, a real-time PCR (qPCR) was performed [34]. The pair of primers sense strand 5′ TGT CGC TTG CAG ACC AGA TG 3′ and antisense strand 5′ GCA TCG CAG GTG TGA GCA C 3′, which amplify a 120 bp fragment, were used to detect and quantify the parasite’s target DNA polymerase (*L. infantum* single-copy gene). Reactions were performed using a Sybr green I (Bryt Green^®^, Promega) fluorescence system in an ABI Prism 7500 Sequence Detection System (Applied Biosystems, Waltham, MA, USA). The number of copies of the parasite’s DNA was determined by a linear regression using the Ct values obtained by the standard curve constructed through serial dilutions (10×) of a fragment containing 10^5^ to 10^0^ copies (efficiency 96.0%; r^2^ = 0.99). The results are expressed by the number of copies of the parasite’s DNA per milligram (mg) of tissue.

### 2.11. Statistical Analysis

Statistical analyses were performed using the GraphPad Prism 8.0 software. The Shapiro–Wilk test was used to confirm the normality of the data. The data were plotted as absolute values or mean/median ± standard deviation (SD)/standard error of means (SEM) of two independent experiments. Analysis of variance (one-way ANOVA) was performed, followed by Tukey’s multiple comparison test or Kruskal–Wallis followed by Dunn’s multiple comparison test to determine the significant differences between groups. The statistical significance was set at *p*-value < 0.05.

## 3. Results

### 3.1. Inflammatory Cells Infiltrating in Mouse Skin

**Figure 1:**
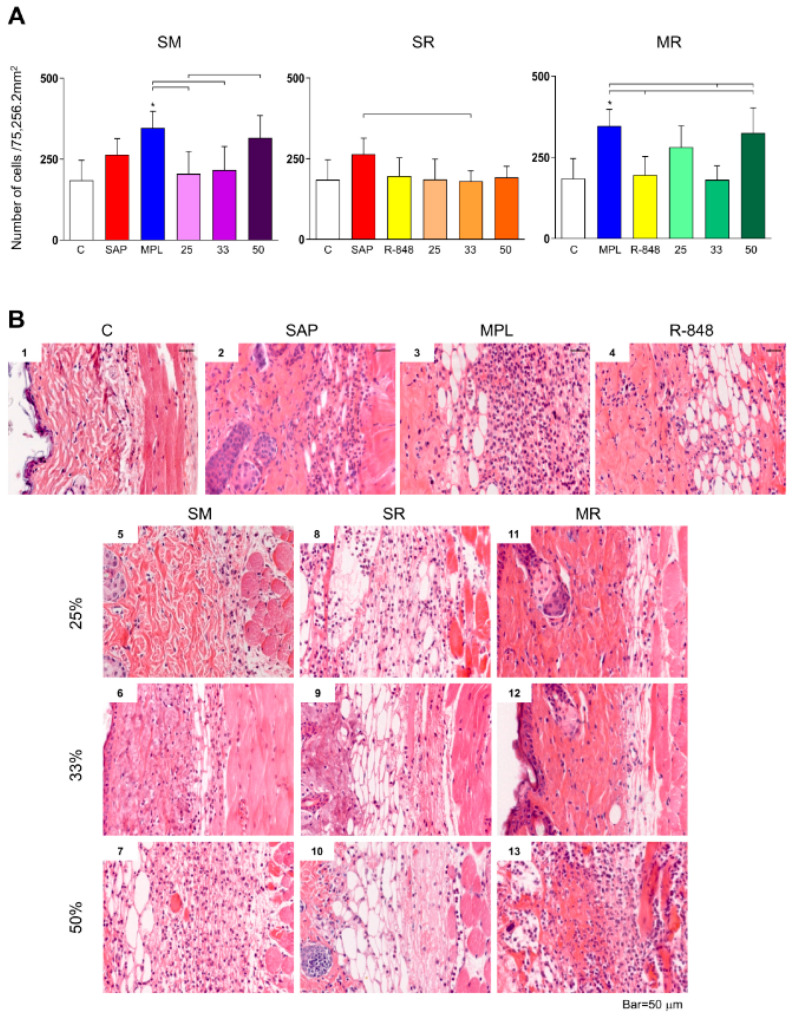
Inflammatory cells infiltrate evaluation in the BALB/c mouse skin 48 h after sensitization with the adjuvants isolated or combined into adjuvant systems (*n* = 12/group). (**A**) Inflammatory cells infiltrating in the dermis/epidermis were expressed by the number of cells per 75,256.2 mm^2^. The data analyses were performed by one-way Anova followed by Tukey’s multiple comparison test and expressed as absolute values ± SD. The significant differences are indicated by connector lines for comparison between the groups (*p*-value < 0.05) and by (*) for comparison with the control group (C). (**B**) Representative photomicrographs of the inflammatory cells infiltrated in the BALB/c mouse skin 48 h after sensitization with the adjuvants isolated or combined into adjuvant systems. (1) Control group, (2) SAP, (3) MPL, (4) R-848, (5) SM25, (6) SM33, (7) SM50, (8) SR25, (9) SR33, (10) SR50, (11) MR25, (12) MR33, and (13) MR50. Slides shown at 40× magnification. Hematoxylin–eosin. Bar = 50 μm.

We found an increase in the number of inflammatory cells in the MPL group when compared to the control group, R-848 group, as well the SM25, SM33, and MR33 adjuvant system groups. Additionally, sensitization with the SM50 adjuvant system significantly increases the inflammatory cells infiltrating the mice’s skin in comparison with the SM25 sensitization. Sensitization with SAP significantly increases the inflammatory cells in comparison with the SR33 adjuvant system group (Figure 1A,B). The mice did not exhibit any macroscopic alteration at the inoculum site (Figure 1B).

### 3.2. Sensitization with the SR Adjuvant System Promotes a Polymorphonuclear Cells Influx to the Inoculum Site

**Figure 2:**
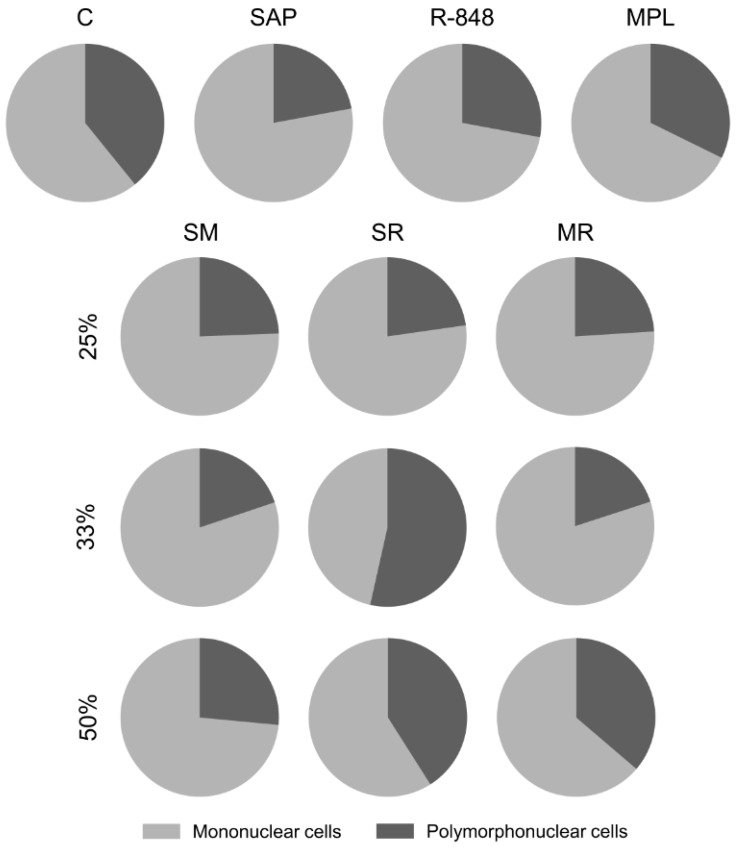
Differential leukocyte count in the BALB/c mouse skin 48 h after sensitization with the adjuvants isolated or combined into adjuvant systems (*n* = 12/group). Percentage values of mononuclear (light gray) and polymorphonuclear (dark gray) cells were displayed on pie charts.

To determine the cell profile recruited to the inoculum site, we evaluate the percent number of mononuclear (lymphocytes and macrophages) and polymorphonuclear (eosinophils, basophils, and neutrophils) cells (Figure 2).

Sensitization with SAP adjuvant showed a decrease in the percentage of polymorphonuclear cells, mainly segmented neutrophils, in comparison with the saline control group. Concerning mononuclear cells, the SM50 adjuvant system group presented macrophages increase when compared to the control and SAP adjuvant groups, as well as the SM25 and SM33 systems. We found that all MR adjuvant system groups stimulated macrophage recruitment at the inoculum site when compared to control and R-848 adjuvant groups. Additionally, the MR25 induced a macrophage increase in comparison with the MPL adjuvant group. In contrast, only sensitization with SR33 leads to a significant reduction in mononuclear cells, especially lymphocytes, at the inoculum site when compared with the SAP and R-848 adjuvants groups; meanwhile, there was a significant increase in polymorphonuclear cells when compared with the SAP and R-848 groups (Figure 2).

### 3.3. SM Adjuvant System-Induced Chemokine Production in Mouse Skin

**Figure 3:**
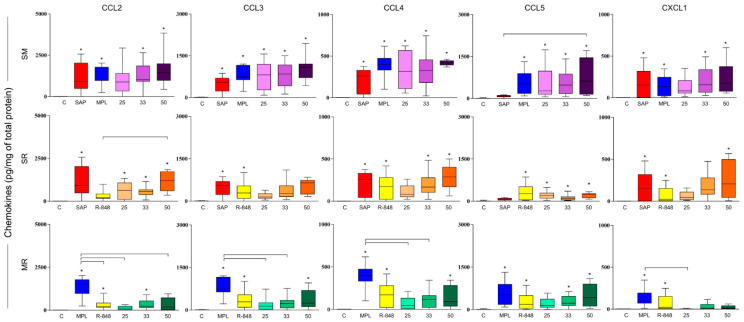
Chemokines measurement (CCL2, CCL3, CCL4, CCL5, and CXCL1) in the BALB/c mouse skin 48 h after sensitization with the adjuvants isolated or combined into adjuvant systems (*n* = 12/group). Chemokine production was expressed by picograms per milliliter (pg/mL) of total protein. The data analyses were performed by Kruskal–Wallis test and expressed as mean/median ± SD. The significant differences are indicated by connector lines for comparison between the groups (*p*-value < 0.05) and by (*) for comparison with the control group (C).

After 48 h of sensitization, all adjuvants isolated significantly increase the levels of CCL2, CCL3, CCL4, CCL5, and CXCL1 chemokines when compared to the mice control group, except for the R-848 and SAP groups concerning CCL2 and CCL5 chemokine, respectively (Figure 3). 

Regarding CCL2 chemokine, SM adjuvant system groups that received 33% or 50% of each adjuvant total dose exhibited a significant increase in comparison with the mice that received sterile saline. This finding was also observed when mice received the MR33 adjuvant system (Figure 3). Sensitization with the SR adjuvant system in all doses showed increased levels of CCL2 chemokine when compared to the control group, and SR50 also when compared to the R-848 adjuvant group (Figure 3).

Sensitization with all doses of SM adjuvant system triggered an increase in CCL3 and CCL4 levels in comparison to the saline control group. Additionally, the MR50 adjuvant system showed higher levels of the CCL3 chemokine when compared to the saline control group (Figure 3). SR adjuvant system groups that received 33% or 50% of each adjuvant total dose, as well as those who received MR50, showed a significant augment of CCL4 chemokine in comparison with the control group (Figure 3).

All adjuvant systems at the above-mentioned doses showed higher levels of CCL5 chemokine in comparison with the control group, except for MR25. Furthermore, sensitization with the SM50 adjuvant system also presented a significant increase in comparison with the group that received SAP adjuvant only (Figure 3).

Finally, we found increased levels of CXCL1 chemokine after sensitization with the SM33, SM, and SR adjuvant groups that received 50% of each adjuvant total dose when compared to mice that received sterile saline (Figure 3).

### 3.4. Sensitization with Adjuvant Systems Led to Pro-Inflammatory Microenvironment in Mouse Skin

**Figure 4:**
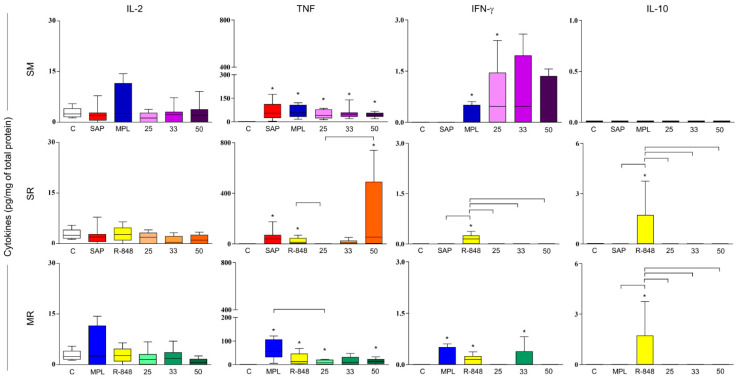
Cytokine measurement (IL-2, TNF, IFN-γ, and IL-10) in the BALB/c mouse skin 48 h after sensitization with the adjuvants isolated or combined into adjuvant systems (*n* = 12/group). Cytokine production was expressed by picograms per milliliter (pg/mL) of total protein. The data analyses were performed by Kruskal–Wallis test and expressed as mean/median ± SD. The significant differences are indicated by connector lines for comparison between the groups (*p*-value < 0.05) and by (*) for comparison with the control group (C).

To the IL-2 cytokine, no differences were observed among the groups (Figure 4). All adjuvants isolated induce increased levels of pro-inflammatory cytokines (TNF and IFN-γ) when compared with the mice control group, except for the SAP group concerning IFN-γ cytokine. Sensitization with SM25 stimulated a significant increase in TNF and IFN-γ; meanwhile, we found that sensitization with SM33 and SM50 only increased TNF cytokine in comparison with the mice that received sterile saline (Figure 4). We observed a significant augmentation in TNF levels in all adjuvant system groups that received 50% of each adjuvant total dose in comparison with the control group. Additionally, the SR50 adjuvant system group was significantly different from the SR25 group (Figure 4). Regarding IFN-γ, our data demonstrate an increase in that cytokine in the MR33 adjuvant system group when compared with the control group (Figure 4). Lastly, we observed higher levels of IL-10 in mice that received the R-848 adjuvant only when compared to control, SAP, MPL, and SR and MR adjuvant systems groups (Figure 4).

**Figure 5:**
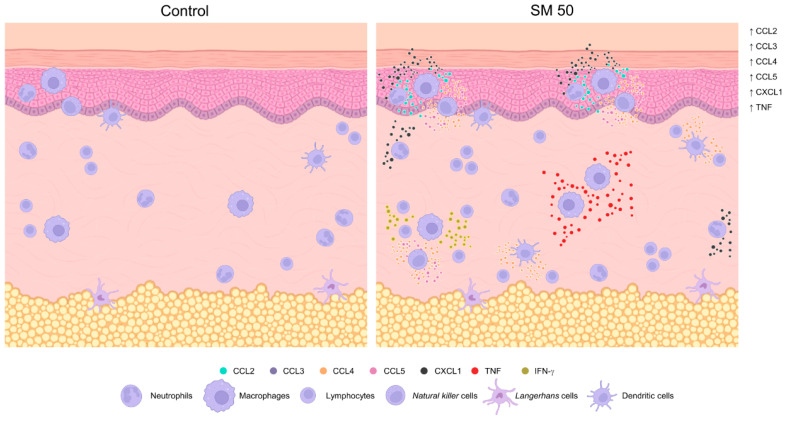
Illustrative figure of the main differences observed in the BALB/c mouse skin 48 h after sensitization with the adjuvant system SM50 (saponin plus monophosphoryl lipid A; the group that received 50% of each adjuvant total dose) in comparison with control group. Figure developed by the authors using the BioRender (available on https://biorender.com accessed on 15 December 2022).

The sensitization with SM adjuvant system with 50% of each adjuvant total dose (SM50) was able to induce enhanced inflammatory cell infiltrates, especially with macrophages recruitment, high levels of CCL2, CCL3, CCL4, CCL5, CXCL1 chemokines, and production of TNF cytokine. Therefore, SM50 was chosen to continue investigation throughout the study (Figure 5).

### 3.5. Immunization with LBSM50 Stimulated Expressive Pro-Inflammatory Cytokines Production

**Figure 6:**
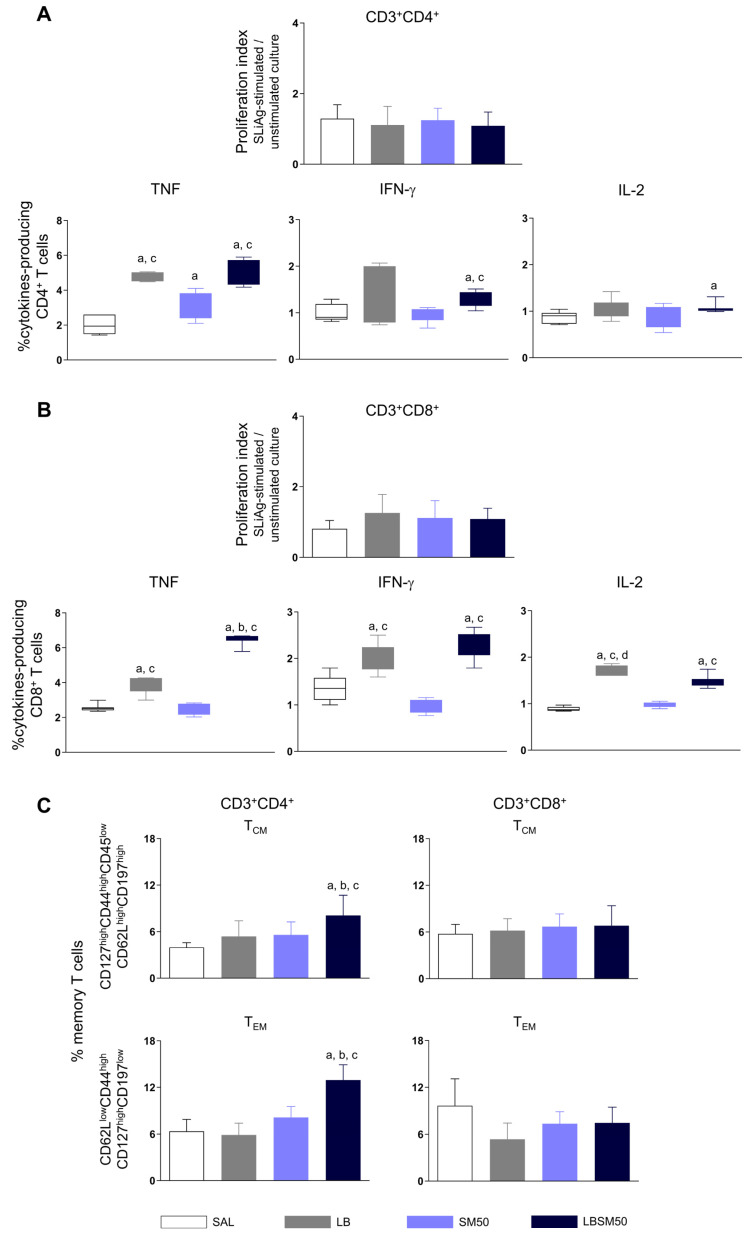
T lymphocytes proliferation, intracellular cytokine production, and memory T cells induced by immunization and challenge with *L. (L.) infantum* in BALB/c mice (*n* = 10/group). Proliferation index of CD4^+^ (**A**) and CD8^+^ (**B**) T cells obtained by the SLiAg-stimulated culture/unstimulated culture ratio. Intracellular cytokines-producing CD4^+^ (**A**) and CD8^+^ (**B**) T cells, and central and effector memory CD4^+^ and CD8^+^ T cells, and (**C**) frequency in SLiAg-stimulated culture. The data analyses were performed by one-way Anova or Kruskal–Wallis and expressed as mean/median ± SD. The significant differences are indicated by the letters “a”, “b”, “c” and “d” for comparison with the control group (SAL), *L. (V.) braziliensis* antigen (LB), and adjuvant group saponin plus monophosphoryl lipid A (SM50), respectively (*p*-value < 0.05).

No significant T cell proliferation was found independent of the immunization that mice received (Figure 6A,B).

Animals immunized with LB antigen only or LBSM50 showed an increase in the frequency of CD4^+^ T cells producers of TNF in comparison with the saline control group as well as the adjuvant system group (Figure 6A). Regarding the IFN-γ-producing CD4^+^ T cells, LBSM50 when compared to control and adjuvant system groups (Figure 6A). Mice that received LBSM50 had increased IL-2-producing CD4^+^ T cells in comparison with the saline control group. (Figure 6A).

We found an enhancement in the frequency of TNF-producing CD8^+^ T cells in immunized mice with LB antigen only and LBSM50 when compared to the saline control and adjuvant system groups (Figure 6B). Additionally, the LBSM50 group was significantly higher than the LB antigen group. We emphasize the expressive increase in TNF level being two and a half-fold higher than the saline control group (Figure 6B). Immunization with LB antigen only or LBSM50 led to an increase in the frequency of IFN-γ-producing CD8^+^ T cells in comparison with the control and adjuvant system groups (Figure 6B). We observe a significant increase in IL-2 levels in LB antigen and LBSM50 groups in comparison with the control and adjuvant system groups. Additionally, the LB group was significantly different from the LBSM50 group (Figure 6B).

### 3.6. Immunization with LBSM50 Induced Differentiation in Memory CD4^+^ T cells

Memory T cell responses are essential to generate long-term protection against *Leishmania* infection. Accordingly, we evaluated the specific CD4^+^ and CD8^+^ T cells combined with markers related to memory T cells phenotype, allowing the definition of central (CD127^high^CD44^high^CD45^low^CD62L^high^CD197^high^) and effector memory T cells (CD62L^low^CD44^high^CD127^high^CD197^low^). We observed a significant increase in the frequency of cells expressing the central as well effector memory CD4^+^ T cells phenotype in immunized mice with the LBSM50 when compared to the mice that received sterile saline, adjuvant system and LB antigen only (Figure 6C). Notwithstanding, after immunization with the LBSM50 no difference was found when we evaluated central or effector memory CD8^+^ T cells (Figure 6C).

### 3.7. Immunization with LBSM50 Elicited Partial Protection against Leishmania Infection

**Figure 7:**
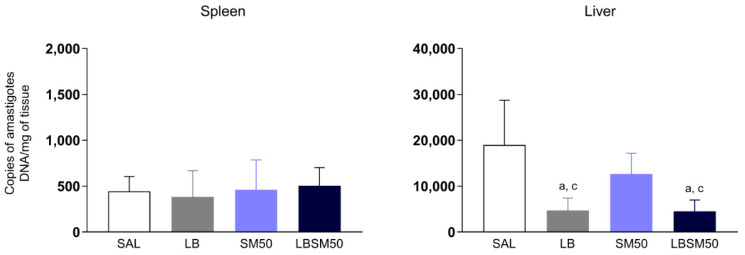
Parasite load in immunized BALB/c mice’s spleen and liver 28 days post-challenge with *L. (L.) infantum* (*n* = 10/group) expressed in copies of amastigotes DNA per milligram (mg) of tissue. The data analyses were performed by one-way Anova and expressed as mean ± SD. The significant differences are indicated by the letters “a” and “c” for comparison with the control group (SAL) and adjuvant group saponin plus monophosphoryl lipid A (SM50), respectively (*p*-value < 0.05).

The vaccine efficacy was performed by the parasite load quantification in mice’s spleen and liver. No difference was found in the splenic parasite load of the immunized mice with LBSM50 and the control groups. However, in the liver, there was a decreased parasite load when mice received LB antigen or LBSM50 in comparison with the saline and adjuvant system control groups (Figure 7).

## 4. Discussion

We evaluated the adjuvants SAP, MPL, and R-848 isolated and combined in a lower dose (SM, SR, and MR) equal to 25%, 33%, and 50% of each adjuvant total dose in the skin of mice sensitized by intradermal route. A cutaneous swelling at the inoculum site was a sign of successful intradermal administration. Sensitization with the different adjuvant systems appeared to be safe for administration.

Sensitization with MPL adjuvant isolated induced an increase in cell recruitment in the mouse skin, as shown previously [7]. MPL is a TLR4 agonist, a strong inductor Th1-type immune response, a promoter of APCs activation, and a stimulator of monocytes and dendritic cell migrations to the inoculum site [7,35,36]. Additionally, SM and MR33 groups also lead to local cell recruitment, which is important since it directs the immune response. The availability of cells capable of recognizing the vaccine antigens and developing an appropriate response increases the potential for vaccinal success.

Only the sensitization with the SR33 adjuvant system promoted a replacement of mononuclear by polymorphonuclear cells recruited to the inoculum site. We suggest that the mononuclear cell reduction, especially lymphocytes, and polymorphonuclear cell increase may be a result of the sensitization-related injury. As is known, after a tissue injury or immune stimulation, neutrophils are the first cells to arrive at the site [7,37,38]. Moreover, neutrophils are relevant producers of chemokines and cytokines involved in the recruitment and activation of other cells, as M1 or M2 macrophages, for example [39]. Conversely, SM50 increased macrophage recruitment.

An intrinsic factor that directly influences cell recruitment profile is the pattern of chemokine production induced by the adjuvants alone or combined as an adjuvant system in the inoculum site. Chemokines are able to interact with more than one receptor present, mostly in leucocytes, triggering the recruitment of these cells inside of tissues [40]. All adjuvants evaluated are TLR’s agonists, which usually are responsible for the antigen recognition and trigger a signaling pathway that leads to chemokine production and, hence, immunocompetent cell recruitment [41,42]. CCL2, CCL3, CCL4, CCL5, and CXCL1 chemokine production was induced by SR33 and SR50. Additionally, MR33 and MR50 groups presented increased levels of CCL2, CCL3, CCL4, and CCL5 chemokines. Moreover, significantly high levels of all chemokines were found only with SM33 and SM50. CXCL1 chemokine production is related to neutrophils recruitment to the site of injection, which is a relevant cell group responsible for setting up the first line of defense against infections [38]. The CCL2, CCL3, CCL4, and CCL5 chemokines are closely correlated with migration of monocytes from blood to tissue, macrophage recruitment, and cell proliferation after a stimulus [43,44,45,46,47,48]. Supported by these previous studies, we can suppose that the adjuvant systems were able to promote an innate immune response that may induce a recruitment of cells to the inoculum site, creating a favorable microenvironment to antigen uptake and presentation.

As expected, the adjuvant system contributes, at least, to an additive effect on immune response. Thus, we evaluated cytokine production to analyze whether the cells were activated on the site of sensitization. Except for the group that received sterile saline, we observed an induced cytokine production in all groups, mainly Th1 (TNF and/or IFN-γ). Pro-inflammatory cytokines secreted by resident and recruited cells modulate this microenvironment and stimulate the activation of APCs that will further migrate to the draining lymph nodes and, hence, improve the chances of a successful vaccination. Regarding the production of IL-10 cytokine, only R-848 stimulated their production as previously described [49,50]. It is worth pointing out that the SM adjuvant system has the ability to induce production of cytokines even in low doses, especially when compared with each adjuvant alone. Similarly, previous studies found a quick activation of DCs by using an adjuvant system (AS01) produced by *GlaxoSmithKline* (i.e., QS-21 and MPL-A in a liposomal emulsion) [16,51,52]. Our findings partially resemble those results despite our option to use the crude SAP instead of a purified fraction and the absence of liposomes in the system composition. 

As is known as being a successful strategies for vaccines [53], employing attenuated *Leishmania* strains generates a long-lasting protective immune response [54,55]. The cross-protection of *L*. (*V*.) *braziliensis* antigen against *L*. (*L*.) *infantum* infection was already described [30,56,57], which justifies our decision to associate the adjuvant system SM50 with the crude *L*. (*V*.) *braziliensis* antigen followed by the evaluation of the specific immune response triggered and the efficacy against *L*. (*L*.) *infantum* in BALB/c mice.

The immunization using LBSM50 induced differentiation in central and effector memory CD4^+^ T cells. These cells were previously characterized as correlates of protection against visceral leishmaniasis due to their role in the immune response, i.e., renewing memory cells while effector memory cells migrate to infection sites and eliminate parasites [58]. Central and effector memory cells are essential to parasite elimination and disease control [59,60]. Additionally, central memory T-cells play an important role in the immune response due to the renewing of memory cells while effector memory cells migrate to the infection site and help to eliminate the parasite [61]. Effector memory T cells express low or no CCR7 chemokine receptors (CCR7) that are responsible for the cell migration to secondary lymphoid organs [33]. This subpopulation presents migration receptors that lead these cells to inflammatory tissues for playing their immediate effector function [33,62]. It is worth mentioning that increased parasite load in the liver must probably trigger the intense inflammatory process that is recruiting these cells. Regarding cytokines, Darrah et al. [63] demonstrated a protective profile linked to inducing effector memory T cells Th1-type for the first time in a vaccinal study against Leishmaniasis using this model. Therefore, our data agree with other studies, which reinforce using an adjuvant system based on combining adjuvants in order to promote a potent immune response for increasing the protective effect of a possible vaccine candidate [64].

Lastly, we evaluated the parasite load in the spleen and liver of mice. The low parasite load observed in the mouse spleen can be explained by the kinetics of the *L.* (*L.*) *infantum* infection with the strain MCAN/BR/2008/OP46. As described by Reis et al. [34] and Rodrigues et al. [65], there was a high initial hepatic parasite load (acute infection) within the first two weeks (resistance to infection and maybe resolving infection), while there was low and almost absent splenic parasite load until the fifth week (parasite persistence). Over the years, the liver was more recognized as a crucial immune organ associated with immunological memory and important action in the acute stage of infections [65]. We found no difference in splenic parasite load, but the hepatic parasite load was decreased after immunization with LBSM50. Moreover, we observed cross-protection with the heterologous *L.* (*V.*) *braziliensis* antigen against experimental challenge with *L.* (*L.*) *infantum*. Previous studies already showed similar findings, i.e., partial protection against *L*. (*L*.) *major* and *L*. (*V*.) *braziliensis* infection in BALB/c mice vaccinated with *L*. (*L*.) *amazonensis* antigen [66,67]. Furthermore, whether homologous or heterologous, the protective immune mechanisms and the efficacy against Leishmania parasites remain unknown [53].

## 5. Conclusions

SM50, when combined with *Leishmania* (*V.*) *braziliensis* antigen, increases TNF and IFN-γ, which generate central and effector memory CD4^+^ T cells. Moreover, our vaccine candidate conferred partial protection, due to a reduced parasite load in the liver, based on an induced rapid immune response directed to this organ. We highlight the sensibilization with SM50 that induces an enhanced inflammatory cell infiltrate, especially with macrophages recruitment, high levels of CCL2, CCL3, CCL4, CCL5, CXCL1 chemokines, and production of TNF cytokine.

## Data Availability

The materials, data, and any associated protocols that support the findings of this study are available from the corresponding authors upon request.
